# Predictors of Infarct Growth in Patients with Large Vessel Occlusion Treated with Endovascular Therapy

**DOI:** 10.3389/fneur.2017.00574

**Published:** 2017-10-30

**Authors:** Claus Z. Simonsen, Irene K. Mikkelsen, Sanja Karabegovic, Pia Kjaer Kristensen, Albert J. Yoo, Grethe Andersen

**Affiliations:** ^1^Department of Neurology, Aarhus University Hospital, Aarhus, Denmark; ^2^Center for Functionally Integrative Neuroscience, Aarhus University Hospital, Aarhus, Denmark; ^3^Neuroradiology, Aarhus University Hospital, Aarhus, Denmark; ^4^Clinical Epidemiology, Aarhus University Hospital, Aarhus, Denmark; ^5^Neuroendovascular Service, Texas Stroke Institute, Dallas, TX, United States

**Keywords:** acute ischemic stroke, endovascular therapy, infarct growth, magnetic resonance imaging

## Abstract

**Introduction:**

Endovascular therapy (EVT) is now evidence based in anterior circulation stroke caused by large vessel occlusion. Outcome is related to infarct size, but data on predictors of infarct growth is limited. We analyzed our cohort of EVT treated patients primarily selected by magnetic resonance imaging (MRI) to examine predictors of infarct growth and the association between infarct size and outcome.

**Methods:**

We identified 342 patients with anterior circulation stroke from 2004 to 2014 in our prospectively collected EVT database. Baseline infarct size was available for 281 (measured by MRI) while final infarct size was available for 312 patients. Functional outcome was defined by modified Rankin Score (mRS) after 90 days and good outcome was defined as mRS 0–2. Predictors of infarct growth were examined by regression analysis.

**Results:**

Successful reperfusion [odds ratio (OR) 0.17, 95% confidence interval (CI) (0.09–0.33)] was the strongest predictor of reduction of infarct growth. Receiving intravenous thrombolysis and a short time span from symptom onset to scanning also reduced infarct growth. Occlusion of the internal carotid artery (ICA) intracranially predicted infarct growth (OR = 7.29, 95% CI: 2.36–22.53). EVT under general anesthesia and having a NIHSS between 10 and 15 were also associated with infarct growth.

**Discussion:**

Failure of reperfusion resulted in an average infarct growth of approximately 50 ml. Lack of reperfusion generally results in a poor outcome likely due to infarct growth. Occlusion of the intracranial ICA and EVT under general anesthesia predicted infarct growth, while successful reperfusion, getting intraveneous thrombolysis, and a short time span from onset to scan protected against growth. A median infarct size of 52 ml best discriminates between a good and a bad outcome.

## Introduction

For large vessel occlusion (LVO), endovascular therapy (EVT) is now evidence-based treatment, with five randomized trials demonstrating a beneficial effect (1–5). The goal of EVT is to prevent growth of the infarct core into the threatened penumbra. Given the impact on outcome of final infarct volume (6, 7), factors that predict infarct growth are of great importance in acute stroke therapies. In this paper, we examine factors associated with infarct growth in a population of EVT patients imaged with magnetic resonance imaging (MRI).

Most centers use computed tomography (CT) as imaging selection prior to therapy and cannot, therefore, reliably determine baseline infarct size, a prerequisite for evaluating growth. Diffusion weighted imaging (DWI) is the best clinically available tool to measure the volume of infarcted tissue in the hyperacute phase (8). Since we primarily scan all acute stroke patients with LVO with MRI both pre- and posttreatment, we are able to measure infarct growth on an individual patient level. This information provides a unique perspective in evaluating the question of factors that affect infarct growth in this population.

## Materials and Methods

Data from all consecutive patients treated with EVT for anterior circulation stoke at our center were collected in the period 2004–2014. Our selection criteria for EVT were an acute ischemic stroke with occlusion of a large vessel. The occlusions treated included extracranial internal carotid artery (ICA), intracranial ICA occlusion (ICA-T), and the first or second branch of the middle cerebral artery (MCA). A cervical ICA occlusion together with any intracerebral occlusion is referred to as a tandem occlusion. We excluded patients with infarcts larger than a third of the MCA territory estimated by visual inspection. The patients had to be in a good medical condition and independently living, although comorbid conditions were not specified. The treatment window was 6 h from symptom onset; however, 25 (7%) patients had unknown onset and were potentially treated outside of the 6 h window due to a favorable scan. In addition, seven patients (2%) had DWI lesion larger than a third of the MCA territory, but were treated because of patient specifics (e.g., young age).

Imaging assessment at presentation was made using MRI DWI whenever possible. CT was chosen if the patient had a contraindication to MRI (e.g., pacemaker), if the patient was too unstable (e.g., vomiting, respiratory concerns) or if the patient could not fit within the MRI scanner (e.g., obesity, severe cervical kyphosis.) The patients were treated with intravenous (IV) tissue plasminogen activator (tPA) if there was no contraindication and taken directly for EVT without waiting for a clinical response. From 2004 to January 2010, treatment was limited to day time, but from January 2010, there was 24/7 coverage.

Reperfusion was assessed by the modified thrombolysis in cerebral infarction (mTICI) score where 0 denotes no reperfusion, 1 minimal flow past occlusion but no distal reperfusion, 2a reperfusion of <50% of the ischemic territory, 2b >50% reperfusion, and 3 signifies full reperfusion of the ischemic territory. Successful reperfusion was defined as mTICI 2b or 3 post EVT. Clinical outcome was assessed at 90 days using the modified Rankin Scale (mRS); good outcome was defined as 90-day mRS 0–2 (i.e., functional independence).

Infarcts were outlined for the purpose of volume determination using an in-house semiautomatic program on the acute DWI scan, and on the 24-h posttreatment (±6 h) follow-up CT- or DWI scan, whichever was present. Delineation of the infarct sizes was done by a consensus read by the neurologist (Claus Z. Simonsen) and a neuroradiologist (Sanja Karabegovic) blinded to the outcome of the patient.

We treated 342 patients with EVT for anterior circulation stroke in the time period. For follow-up infarct imaging, 15 scans were acquired on an older scanner from which images could not be analyzed. Twelve follow-up scans were not done, either because the patient was discharged early or was transferred to another department. Two patients died before follow-up imaging could be done. One scan had such poor quality, that it was not possible to accurately delineate the infarct border. Final infarct data were available for 312 (91%) patients, of which, 195 were MRI scans and 117 CT scans. It was possible to perform MRI acutely in 281 patients (82%). In these 281 patients, we examined infarct growth and the predictors of growth. Growth was defined as CT- or DWI lesion volume on the 24-h scan subtracted by the DWI lesion seen on the acute scan before EVT.

The study was conducted with the approval of the local Ethical Committee of Central Denmark Region (Journal number 1-16-02-397-12).

### Statistical Analysis

Standard descriptive statistics were used to summarize the baseline clinical and imaging variables. The Chi-square test was used for categorical variables. The Kruskal–Wallis test was used to compare continuous variables. A receiver operator characteristic (ROC) analysis was done to find the infarct size that best predicted non-independence.

Second, we used univariate logistic regression to identify potential predictors of growth above 50 ml. To evaluate the robustness of the dichotomization of infarct growth, we also performed the analysis with ordered logistic regression with infarct growth categorized into three and four groups.

Third, predictors were included in a multiple logistic regression to examine whether all predictors remained independently associated with infarct growth. We replicated the analysis with a categorization of infarct growth using logistic regression. Analyses were performed using STATA (version 12.0, StataCorp LP, College Station, TX, USA) and MedCalc software (version 14.12.0, Ostend, Belgium).

## Results

For the entire cohort of 312 patients, median age was 67 years [interquartile range (IQR) 59–75], NIHSS 17 (IQR 13–20), time from onset to scan 115 min (IQR 81–180 min), and time from scan to groin puncture was 98 min (IQR 67–140 min). Procedure time for the 257 patients who achieved reperfusion was 55 min (IQR 33–96.25 min).

**Figure 1:**
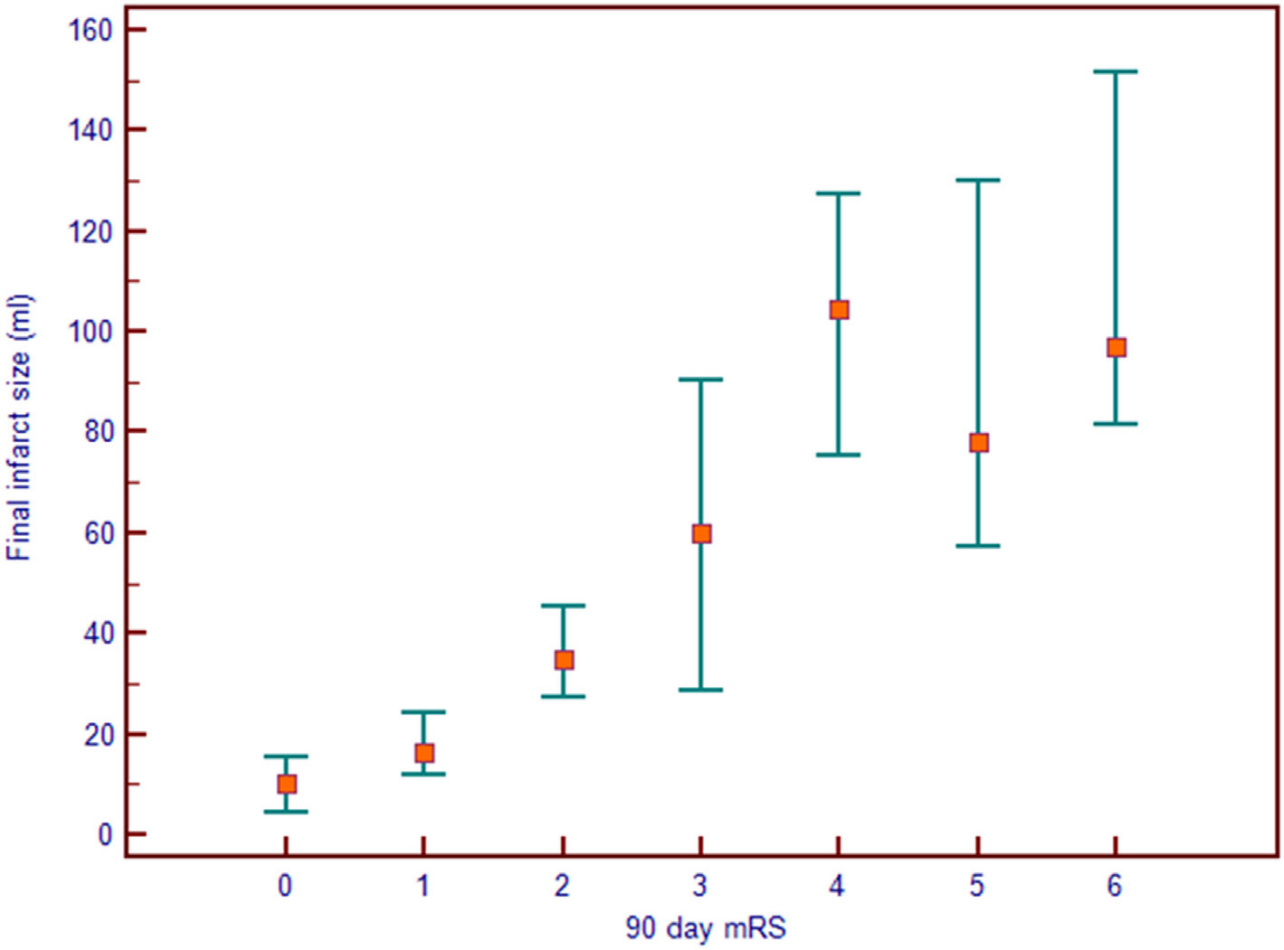
Final infarct size seen on the 24-h magnetic resonance imaging or computed tomography scan seen as a function of the 90-day modified Rankin Score (mRS). The median infarct is shown with a square and the markers denote 95% confidence intervals.

**Figure 2:**
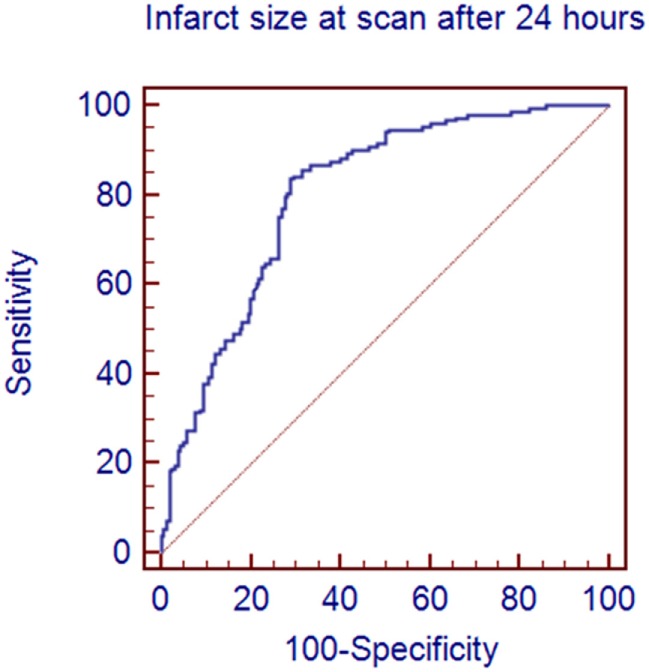
A receiver operator characteristics analysis showing the sensitivity and specificity of predicting a good outcome on the basis of the size of the infarct on the 24-h scan. An infarct <52 ml predicted a good outcome with a sensitivity of 84.3% and a specificity of 70.4%.

Figure 1 demonstrates a clear association between final infarct size and outcome for the 312 patients with a 24-h scan. Lower final infarct size is related to lower mRS. Final infarct sizes lower than 50 ml generally result in mRS 0–2, i.e., independent living. The median final infarct sizes for the mRS strata (0–6) are provided in Table 1. The odds ratio (OR) of achieving a good outcome if final infarct was <50 ml was 12.58 [95% confidence interval (CI) 7.27–21.77]. An ROC analysis on final infarct size and its relation to a good outcome showed that a final infarct of 51.6 ml was the best discriminator for a good outcome (mRS 0–2, sensitivity 84.3%, specificity 70.4%), see Figure 2. With final infarct volume larger than 90 ml, the specificity of a poor outcome was 95% (Table 2).

**Table 1:** Median final infarct sizes with interquartile ranges 24 h after the stroke in the seven modified Rankin scale (mRS) groups.

90-day mRS	Number of patients	Final infarct (ml)
0	29	10.3 (3.1–22.6)
1	65	16.5 (7.0–33.7)
2	59	34.9 (14.3–72.7)
3	42	59.9 (16.6–108.6)
4	62	104.5 (42.3–150.7)
5	21	77.9 (55.6–134.9)
6	34	96.9 (62.7–215.0)

**Table 2:** Specificity of a poor outcome (dependent living or death, i.e., modified Rankin scale 3–6) related to infarct size on the 24-h scan.

Infarct greater than (ml)	Specificity for poor outcome
50	128/153 = 83.7%
60	133/153 = 86.9%
70	135/153 = 88.2%
80	138/153 = 90.2%
90	145/153 = 94.8%

Among the 281 patients for whom both an acute DWI MRI and a follow-up scan with final infarct volumes were available, we found an overall median growth of 19.5 ml (IQR 3.7–66.3 ml). The strongest univariate predictor of reduced infarct growth was successful reperfusion with OR = 0.25, CI: 0.15–0.43. Other significant univariate predictors were level of occlusion (ICA-T occlusion with an OR of 6.23, 95% CI: 2.34–16.60 and extracranial ICA with OR 2.59, 95% CI: 1.18–5.65), having an NIHSS 10–15 (OR = 2.35, 95% CI: 1.29–4.27), EVT performed under general anesthesia (OR = 2.26, 95% CI: 1.19–4.27), and receiving IV tPA (OR 0.47, 95% CI: 0.27–0.85). None of the vascular risk factors (diabetes, smoking, atrial fibrillation, age, or sex) were associated with growth. The time spans “onset to scan” and “scan to groin puncture” did not reach statistical significance in the univariate analysis, but when taking other potential covariates into account, “onset to scan” reached statistical significance (Table 3). All predictors except for extracranial ICA remained independently associated with infarct growth in the multiple regression analysis and the OR did not change noticeably (Table 3). Baseline data are provided in supplementary table.

**Table 3:** Predictors for infarct growth above 50 ml, *n* = 268.

	Growth >50 ml (*n*)	OR_crude_	[95% confidence interval (CI)]	OR_adj_	(95% CI)
Reperfusion					
No (ref)	53% (49/92)				
Yes	22% (39/176)	0.25	(0.15–0.43)	0.17	(0.09–0.33)
IV tissue plasminogen activator					
No (ref)	46% (29/63)				
Yes	29% (59/205)	0.47	(0.27–0.85)	0.40	(0.19–0.84)
Gender					
Women (ref)	29% (31/106)				
Men	35% (57/162)	1.31	(0.77–2.23)	1.06	(0.52–2.15)
Smoker					
No (ref)	34% (63/185)				
Yes	30% (24/81)	0.82	(0.46–1.44)	0.66	(0.32–1.37)
Hypertension					
No (ref)	34% (46/134)				
Yes	31% (42/134)	0.87	(0.52–1.45)	0.79	(0.40–1.58)
Diabetes					
No (ref)	31% (74/238)				
Yes	47% (14/30)	1.94	(0.90–4.18)	1.60	(0.61–4.18)
Atrial fibrillation					
No (ref)	36% (68/187)				
Yes	25% (20/81)	0.57	(0.32–1.03)	0.63	(0.28–1.44)
General anesthesia					
No (ref)	21% (15/72)				
Yes	37% (73/196)	2.26	(1.19–4.27)	2.40	(1.07–5.37)
NIHSS					
<10	24% (25/106)	1.00	(Reference)		
10–15	42% (42/100)	2.35	(1.29–4.27)	2.13	(0.98–4.63)
>15	34% (21/62)	1.66	(0.83–3.31)	1.40	(0.58–3.39)
Location of occlusion					
M1	26% (33/129)	1.00	(Reference)		
Internal carotid artery (ICA) extracranial	47% (16/34)	2.59	(1.18–5.65)	2.41	(0.84–6.87)
ICA-T	68% (15/22)	6.23	(2.34–16.60)	7.29	(2.36–22.53)
M2	14% (3/21)	0.48	(0.13–1.75)	0.26	(0.05–1.42)
Tandem	34% (21/61)	1.53	(0.79–2.95)	1.89	(0.79–4.54)
Time, onset to scan (min)		0.68	(0.45–1.04)	0.52	(0.30–0.88)
Time, scan to groin (min)		0.87	(0.55–1.36)	0.63	(0.35–1.13)
Age (years)		1.01	(0.99–1.02)	1.01	(0.99–1.03)

*Predictors of dichotomized infarct growth in anterior circulation stroke. Odds ratio (OR) describes the risk of growth >50 ml. A number lower than 1 means that the factor protects against growth. NIHSS strata was compared to NIHSS 0–9 as a reference. Lesion location was compared to M1, which was the most prevalent lesion location*.

Looking generally at infarct growth, the median growth among reperfusers (mTICI 2b + 3) and non-reperfusers was 12.2 ml (2.1–44.4) and 57.4 ml (12.7–105.0), respectively (*p* < 0.0001). Among M1 occlusions (*n* = 129), median infarct growth among reperfusers (*n* = 89) was 6.7 ml (0.95–23.07). In the non-reperfusers (*n* = 40), infarcts grew with a median of 58.9 ml (12.8–104.5), *p* < 0.0001.

**Figure 3:**
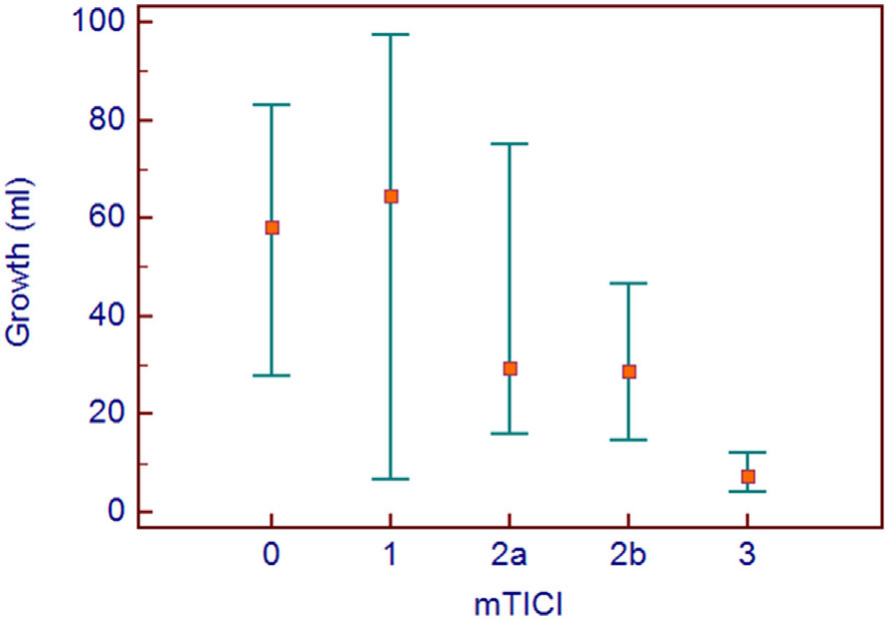
Median growth with interquatile ranges are shown for the modified thrombolysis in cerebral infarction groups.

Since reperfusion was such an important predictor, an analysis of growth related to mTICI score was done. A median growth of about 60 ml was seen in mTICI 0–1, 30 ml in mTICI 2a and 2b, and 7.4 ml in complete reperfusion (mTICI 3). See Figure 3.

We looked at infarct growth from the time span of the first acute DWI scan to reperfusion. We assumed that the infarct stopped growing when reperfusion occurred. We did a regression analysis with growth as a function of time from scan to reperfusion. The coefficient of this line was 0.19, interpreted as a growth of 0.19 ml/min of time elapsing from the first MRI scan to the final infarct was established (*n* = 202). The correlation was significant (*p* = 0.0001). Assuming a brain volume of 1,500 ml and a total number of neurons of 23.3 × 10^9^ (9), this calculates into a loss of 3.0 million neurons per minute.

## Discussion

Knowledge about the relationship between infarct size and long-term outcome is of value in clinical decision making before treatment, and it will facilitate family discussions regarding prognosis after stroke. Our study found a clear association between final infarct size and outcome. The best discriminator between living independently or not was an infarct size of 51.6 ml. Predictors of infarct growth included lack of reperfusion, an ICA-T occlusion, having an NIHSS of 10–15, not receiving IV tPA, EVT under general anesthesia and a long time span from onset to scan. The difference between being reperfused or not resulted in an infarct growth of approximately 50 ml. Reperfusion, therefore, seems to discriminate between a good or a bad outcome as a consequence of the amount of brain tissue that is saved.

Since large final infarcts are related to bad outcomes then it is reasonable to assume that if patients already have a large infarct at presentation, outcome would invariably be poor and that would argue for excluding patients from reperfusion therapy. The ECASS 1 trial on IV tPA showed no effect of treatment, but if patients with larger infarcts (defined as more than a third of the MCA territory) were excluded from the study *post hoc*, there was effect of the treatment (10). In the EPITHET trial, a certain pattern on the perfusion weighted scan with a very large perfusion lesion was termed the “malignant profile” since this pattern was associated with a tendency to intracerebral hemorrhage and a bad outcome (11). Yoo and colleagues found in a cohort of patients treated with EVT that if patients presented with an infarct >70 ml, they all had a poor outcome, although this study was performed in a period where stroke devices were less effective and reperfusion occurred late (6). The same group also found 50 ml as the best discriminator between good and bad outcome (12). A more recent study showed that the maximal infarct volume patients could present with and still achieve a good outcome in 90% of cases was 40 ml (13). A relationship between infarct volume and mRS at 90 days was also found in the DEFUSE study, even though the infarct sizes seemed to be smaller with mRS 3 having a mean infarct size of 40 ml and mRS 4 just below 60 ml (14). We found 60 and 104 ml, respectively.

The sentiment that a lesion volume larger than 70 ml invariable gives a poor outcome is challenged in a paper by Tisserand et al. They find a good outcome on 12/54 (22%) patients that presents with an initial infarct >70 ml and attributes it to DWI reversal (15). In our material, 16 patients presented with an infarct larger than 70 ml and 4 (25%) had a good outcome, but only one had an infarct volume on the 24-h scan that was smaller than on the acute scan.

Even though final infarct size is an important predictor for outcome, there will be outliers with large infarcts that do reasonably well. From Table 2, it can be seen that 8 patients had a final infarct larger than 90 ml and still had a 90-day mRS of 2 or less. The mean age among these patients was 48.5 years, while the 78 patients with a bad outcome had a mean age of 68.5 (*p* = 0.0009). Age was not related to infarct growth, but seems to modify the association between final infarct size and functional outcome. This has also been shown eloquently by Ribo et al. They found the infarct volume discriminating between good and bad outcome to be 49 ml for patients <70 years, 33 ml for patients 70–79 years, and only 15 ml for patients 80 or older (16).

A metaanalysis of the five positive EVT trials showed that there was no benefit of EVT in large infarcts (ASPECTS 0–5) (17) but another analysis from the MR CLEAN study found no interaction of infarct size on the treatment effect (18) meaning that one cannot exclude that there is a treatment effect on the patients presenting with large infarcts. As others, we found that as the infarct volume increases, the specificity of a bad outcome increases, but even at an infarct size of 90 ml, it is not 100%. Research on EVT in these large infarcts is needed.

Reperfusion was the best factor preventing infarct growth. These results are in agreement with those obtained by Man et al. (19) We found that failure of reperfusion resulted in infarct growth of approximately 50 ml. In a comparable analysis, Zaidi and colleagues found a mean infarct volume in the recanalized vs. non-recanalized of 50 vs. 134 ml, respectively (20), while the ESCAPE study found a difference between the recanalized and non-recanalized of only 20.5 ml (21). These studies could only compare the final infarct between reperfusers and non-reperfusers not knowing the initial infarct. The strength of our study is that we can determine infarct growth in the individual patient, since we have acquired MRI DWI on patients before treatment. The level of occlusion (22) has previously been shown to be significantly related to outcome. All retrospective data have shown a relationship with worse outcome and general anesthesia (23). However, recent randomized data draw this into question (24) and the worse outcome seen after general anesthesia could be a result of confounding by indication.

Our study has some weaknesses. For the infarct growth analysis, only patients who had an initial MRI were included. The follow-up scan was ideally an MRI, but in some instances (often in patients with poor medical status), we performed a CT scan. The 24-h CT provided a clear delineation of the infarct margins, thereby minimizing the risk of mismeasurement. But we did not compare patients based on imaging due to the risk of bias introduced by the poorer patients in the CT group. Furthermore, we generally excluded patients with infarcts larger than a third of the MCA territory. Thus, we have no information about infarct evolution in this group of patients. The multivariable regression model may have been overfitted due to the inclusion of a number of potential confounders. However, the model seemed reasonable since the OR estimates did not change substantially from the crude to the adjusted model.

In our analysis of infarct growth over time from the infarct measured at the acute scan to the 24-h scan, we found a growth of 0.19 ml of brain tissue per minute. This translated into a loss of 3.0 million neurons per minute, which is somewhat comparable to the previous calculated 1.9 million neurons per minute (25).

## Conclusion

An infarct size larger than 50 ml significantly increases the risk of an outcome, where the patient is not independently living, but this has to be seen in context of the individual patient. Not achieving reperfusion means an infarct growth of about 50 ml. Lack of reperfusion, ICA-T occlusion, and EVT under general anesthesia were associated with infarct growth.

## Ethics Statement

The study was approved by the Ethical Committee of Central Denmark Region, reference journal number 1-16-02-397-12.

## Author Contributions

All authors contributed to the writing of the manuscript.

## Conflict of Interest Statement

AY: research grants from Penumbra Inc. and Neuravi Inc. CS: lecture fees from Boehringer Ingelheim and Bayer. SK, GA, IM, and PK report no conflicts of interest.

## References

[1] Berkhemer OA Fransen PS Beumer D van den Berg LA Lingsma HF Yoo AJ A randomized trial of intraarterial treatment for acute ischemic stroke. N Engl J Med (2015) 372:11–20. 10.1056/NEJMoa1411587 25517348

[2] Goyal M Demchuk AM Menon BK Eesa M Rempel JL Thornton J Randomized assessment of rapid endovascular treatment of ischemic stroke. N Engl J Med (2015) 372:1019–30. 10.1056/NEJMoa1414905 25671798

[3] Saver JL Goyal M Bonafe A Diener HC Levy EI Pereira VM Stent-retriever thrombectomy after intravenous t-PA vs. t-PA alone in stroke. N Engl J Med (2015) 372:2285–95. 10.1056/NEJMoa1415061 25882376

[4] Campbell BC Mitchell PJ Kleinig TJ Dewey HM Churilov L Yassi N Endovascular therapy for ischemic stroke with perfusion-imaging selection. N Engl J Med (2015) 372:1009–18. 10.1056/NEJMoa1414792 25671797

[5] Jovin TG Chamorro A Cobo E de Miquel MA Molina CA Rovira A Thrombectomy within 8 hours after symptom onset in ischemic stroke. N Engl J Med (2015) 372:2296–306. 10.1056/NEJMoa1503780 25882510

[6] Yoo AJ Verduzco LA Schaefer PW Hirsch JA Rabinov JD Gonzalez RG. MRI-based selection for intra-arterial stroke therapy: value of pretreatment diffusion-weighted imaging lesion volume in selecting patients with acute stroke who will benefit from early recanalization. Stroke (2009) 40:2046–54. 10.1161/STROKEAHA.108.541656 19359641 PMC2709767

[7] Olivot JM Mosimann PJ Labreuche J Inoue M Meseguer E Desilles JP Impact of diffusion-weighted imaging lesion volume on the success of endovascular reperfusion therapy. Stroke (2013) 44:2205–11. 10.1161/STROKEAHA.113.000911 23760215 PMC4396870

[8] Chalela JA Kidwell CS Nentwich LM Luby M Butman JA Demchuk AM Magnetic resonance imaging and computed tomography in emergency assessment of patients with suspected acute stroke: a prospective comparison. Lancet (2007) 369:293–8. 10.1016/S0140-6736(07)60151-2 17258669 PMC1859855

[9] Pakkenberg B Gundersen HJ. Solutions to old problems in the quantitation of the central nervous system. J Neurol Sci (1995) 129(Suppl):65–7. 10.1016/0022-510X(95)00067-C 7595625

[10] Hacke W Kaste M Fieschi C Toni D Lesaffre E von Kummer R Intravenous thrombolysis with recombinant tissue plasminogen activator for acute hemispheric stroke. The European Cooperative Acute Stroke Study (ECASS). JAMA (1995) 274:1017–25. 10.1001/jama.1995.03530130023023 7563451

[11] Davis SM Donnan GA Parsons MW Levi C Butcher KS Peeters A Effects of alteplase beyond 3 h after stroke in the Echoplanar Imaging Thrombolytic Evaluation Trial (EPITHET): a placebo-controlled randomised trial. Lancet Neurol (2008) 7:299–309. 10.1016/S1474-4422(08)70044-9 18296121

[12] Yoo AJ Chaudhry ZA Nogueira RG Lev MH Schaefer PW Schwamm LH Infarct volume is a pivotal biomarker after intra-arterial stroke therapy. Stroke (2012) 43:1323–30. 10.1161/STROKEAHA.111.639401 22426317

[13] Ribo M Tomasello A Lemus M Rubiera M Vert C Flores A Maximal admission core lesion compatible with favorable outcome in acute stroke patients undergoing endovascular procedures. Stroke (2015) 46:2849–52. 10.1161/STROKEAHA.115.010707 26294674

[14] Albers GW Goyal M Jahan R Bonafe A Diener HC Levy EI Relationships between imaging assessments and outcomes in solitaire with the intention for thrombectomy as primary endovascular treatment for acute ischemic stroke. Stroke (2015) 46:2786–94. 10.1161/STROKEAHA.115.010710 26316344

[15] Tisserand M Turc G Charron S Legrand L Edjlali M Seners P Does diffusion lesion volume above 70 mL preclude favorable outcome despite post-thrombolysis recanalization? Stroke (2016) 47:1005–11. 10.1161/STROKEAHA.115.012518 26979862

[16] Ribo M Flores A Mansilla E Rubiera M Tomasello A Coscojuela P Age-adjusted infarct volume threshold for good outcome after endovascular treatment. J Neurointerv Surg (2014) 6:418–22. 10.1136/neurintsurg-2013-010786 23832414

[17] Goyal M Menon BK van Zwam WH Dippel DW Mitchell PJ Demchuk AM Endovascular thrombectomy after large-vessel ischaemic stroke: a meta-analysis of individual patient data from five randomised trials. Lancet (2016) 387:1723–31. 10.1016/S0140-6736(16)00163-X 26898852

[18] Yoo AJ Berkhemer OA Fransen PSS van den Berg LA Beumer D Lingsma HF Effect of baseline Alberta Stroke Program Early CT Score on safety and efficacy of intra-arterial treatment: a subgroup analysis of a randomised phase 3 trial (MR CLEAN). Lancet Neurol (2016) 15:685–94. 10.1016/S1474-4422(16)00124-1 27302238

[19] Man S Aoki J Hussain MS Wisco D Tateishi Y Toth G Predictors of infarct growth after endovascular therapy for acute ischemic stroke. J Stroke Cerebrovasc Dis (2015) 24:401–7. 10.1016/j.jstrokecerebrovasdis.2014.09.004 25499531

[20] Zaidi SF Aghaebrahim A Urra X Jumaa MA Jankowitz B Hammer M Final infarct volume is a stronger predictor of outcome than recanalization in patients with proximal middle cerebral artery occlusion treated with endovascular therapy. Stroke (2012) 43:3238–44. 10.1161/STROKEAHA.112.671594 23160876

[21] Al-Ajlan FS Goyal M Demchuk AM Minhas P Sabiq F Assis Z Intra-arterial therapy and post-treatment infarct volumes: insights from the ESCAPE randomized controlled trial. Stroke (2016) 47:777–81. 10.1161/STROKEAHA.115.012424 26892284

[22] Fischer U Mono ML Schroth G Jung S Mordasini P El-Koussy M Endovascular therapy in 201 patients with acute symptomatic occlusion of the internal carotid artery. Eur J Neurol (2013) 20:1017–24.e1087. 10.1111/ene.12094 23398194

[23] Brinjikji W Murad MH Rabinstein AA Cloft HJ Lanzino G Kallmes DF. Conscious sedation versus general anesthesia during endovascular acute ischemic stroke treatment: a systematic review and meta-analysis. AJNR Am J Neuroradiol (2015) 36:525–9. 10.3174/ajnr.A4159 25395655 PMC8013063

[24] Schönenberger S Uhlmann L Hacke W Schieber S Mundiyanapurath S Purrucker JC Effect of conscious sedation vs general anesthesia on early neurological improvement among patients with ischemic stroke undergoing endovascular thrombectomy: a randomized clinical trial. JAMA (2016) 316:1986–96. 10.1001/jama.2016.16623 27785516

[25] Saver JL Time is brain – quantified. Stroke (2006) 37:263–6. 10.1161/01.STR.0000196957.55928.ab 16339467

