# Supplementary material for: Predictors of Infarct Growth in Patients with Large Vessel Occlusion Treated with Endovascular Therapy
Source: Front Neurol. 2017 Oct 30;8:574. doi: 10.3389/fneur.2017.00574 (PMC5670343; doi:10.3389/fneur.2017.00574)
Supplement: Supplementary file 1 [file data_sheet_1.docx]

Supplementary table.

Baseline data for the cohort of 342 patients with anterior circulation stroke treated with EVT. Numbers are either percentages or medians with interquartile range in parenthesis.

| Age | 67 (59-75) |
| --- | --- |
| NIHSS | 17 (13-20) |
| Percentage women | 39% |
| Percentage left sided strokes | 57% |
| Received IV tPA | 75% |
| Time from last seen well to admission (min) | 79.5 (50-129) |
| Admission to scan (min) | 22 (15-44) |
| Scan to groin puncture (min) | 98 (67-140) |
| Treated under general anesthesia | 75% |
| Successful reperfusion | 65% |
| Occlusion of first branch of Middle Cerebral Artery | 48% |
| Occlusion of second branch of Middle Cerebral Artery | 7% |
| Occlusion of the Internal Carotid Artery in the neck | 13% |
| Occlusion of the Internal Carotid Artery intracranially | 9% |
| Tandem occlusion | 22% |
| Hypertension | 48% |
| Atrial fibrillation | 32% |
| Diabetes | 11% |
| Smoking | 30% |
| NIHSS after 24 hours | 11 (5-18) |
| Rate of good outcome after 90 days | 49% |
